# Down-modulation of functional ventral striatum activation for emotional face stimuli in patients with insula damage

**DOI:** 10.1371/journal.pone.0301940

**Published:** 2024-07-17

**Authors:** Klepzig K., Domin M., von Sarnowski B., Lischke A., Lotze M.

**Affiliations:** 1 Functional Imaging Unit, Institute of Diagnostic Radiology and Neuroradiology, University Medicine Greifswald, Greifswald, Germany; 2 Department of Neurology, University Medicine Greifswald, Greifswald, Germany; 3 Department of Psychology, Medical School Hamburg, Hamburg, Germany; 4 Institute of Clinical Psychology and Psychotherapy, Medical School Hamburg, Hamburg, Germany; Duke University Medical Center: Duke University Hospital, UNITED STATES

## Abstract

Insula damage results in substantial impairments in facial emotion recognition. In particular, left hemispheric damage appears to be associated with poorer recognition of aversively rated facial expressions. Functional imaging can provide information on differences in the processing of these stimuli in patients with insula lesions when compared to healthy matched controls (HCs). We therefore investigated 17 patients with insula lesions in the chronic stage following stroke and 13 HCs using a passive-viewing task with pictures of facial expressions testing the blood oxygenation dependent (BOLD) effect in predefined regions of interest (ROIs). We expected a decrease in functional activation in an area modulating emotional response (left ventral striatum) but not in the facial recognition areas in the left inferior fusiform gyrus. Quantification of BOLD-response in ROIs but also voxel-based statistics confirmed this hypothesis. The voxel-based analysis demonstrated that the decrease in BOLD in the left ventral striatum was driven by left hemispheric damaged patients (n = 10). In our patient group, insula activation was strongly associated with the intensity rating of facial expressions. In conclusion, the combination of performance testing and functional imaging in patients following circumscribed brain damage is a challenging method for understanding emotion processing in the human brain.

## Introduction

A network of various brain areas has been associated with the processing of facial expressions of emotions [1]. This network contains early visual areas in V1-V3 [2] and higher areas relevant for visual recognition [3], the fusiform face area [4], the superior temporal sulcus for the processing of varying aspects of faces relevant for social cognition [5], the ventrolateral prefrontal cortex for emotional processing [6] and a variety of subcortical structures such as the thalamus [7], the superior colliculus [8] and the amygdala [9].

Studies on both healthy participants [1] and on patients with neurodegenerative [10, 11] and cerebrovascular brain disorders [12] additionally highlight the ventral striatum as structure especially relevant for the processing of negative expressions. Among relevant brain structures the insular cortex seems to play a fundamental role being not only relevant for emotion recognition but generally crucial for monitoring one’s own emotional states and that of others, while especially its anterior parts are densely interconnected with other structures highly relevant for affective processing and awareness [13, 14]. Consistently, direct electrocortical stimulation and surgical resection of insula tissue affects emotion recognition [15].

**Fig 1:**
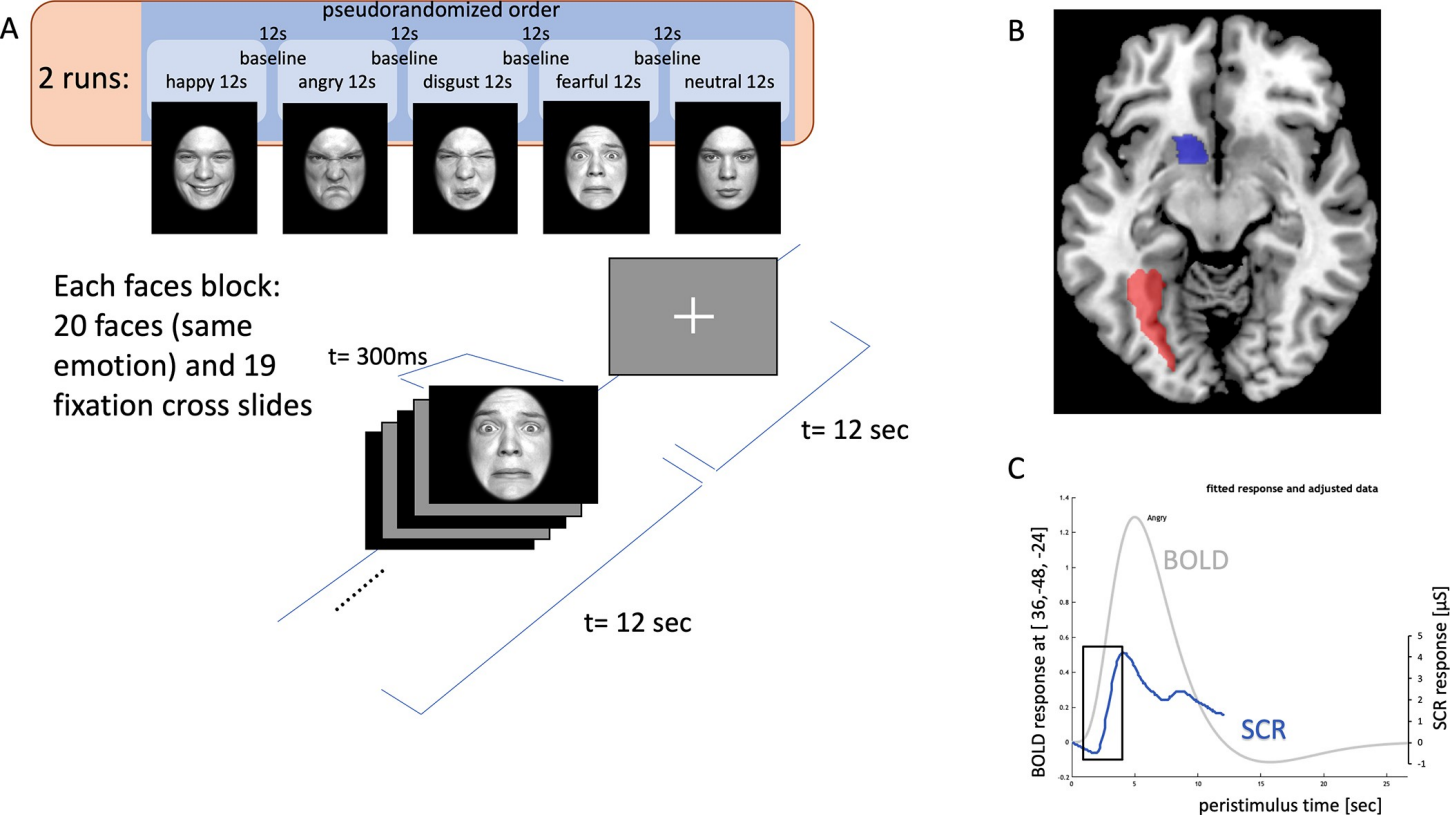
Figure describing the face processing task (A), the selection of left-hemispheric regions of interest (ROIs) for fMRI analysis (z = -10; red = fusiform face area, blue = ventral striatum) using the neurologic convention (right is right) (B), and examples of modulation of blood oxygenation level dependent (BOLD) effect (gray) and skin conductance response (SCR; blue) towards the expression of anger (C).

Likewise, as emotion recognition from faces is frequently impaired in patients suffering from brain damage [16, 17], there is certain evidence that lesions covering the left insula are especially critical [18, 19]. In a recent work of our group emotion recognition impairments in a stroke sample could be referred to lesions of the left insula and were negatively associated with the integrity of associated tracts [20]. As the latter study was part of a comprehensive examination, we also performed a functional imaging study in the same cohort (patients and healthy controls) but focused on stroke patients preselected for showing insula damage in the initial cranial computed tomography and/or the T1 and T2 weighted MRIs. We used a passive-viewing task with pictures showing facial expressions to investigate possible group differences in BOLD response (Fig 1C) between patients and healthy controls (HC). We expected effects for visual processing not only of the earlier face-specific processing representations in the fusiform face area but especially for the ventral striatum as this area receives various insular projections [21]. Possible attentional modulation by emotional facial stimuli of the earlier facial recognition pathway might therefore be unaffected by insula damage [22, 23]. Demographic information and a broad array of cognitive capabilities were assessed to rule out differences between groups which could hamper adequate comparisons. Correlations between BOLD and recognition performance assessed outside the MRI in the same cohort [20] were computed.

Additionally, we also recorded autonomic arousal (assessed as skin conductance response; SCR) which is typically increased during the processing of emotional expressions in healthy populations [24, 25]. In fact, SCR was found to differentiate between facial expressions [24, 26]. In contrast, in patients with insular lesions after stroke reduced arousal have been shown during emotionally evocative stimuli [27, 28], though responses towards facial expressions have been rarely examined in stroke cohorts [29]. However, since insular cortex thickness was found to be positively associated with SCR during facial expressions of emotions in healthy participants [30], we expect to find lowered SCR during face processing in our insula stroke cohort. It is hypothesized that impairments are primarily associated with lesions of the left insula.

## Methods

### Participants

22 Stroke patients with lesions covering the insula were recruited from the stroke unit of the University Medicine Greifswald. Inclusion criteria were: age above 18 and below 90 years, at least five months after stroke onset (chronic stage), and stroke diagnosis based on an initial CT or MRI head scan. Exclusion criteria were: cognitive deficits reported by relatives or physicians, history of additional neurological conditions (neurodegenerative disorders, epilepsy, brain traumas or tumors), and schizophrenia. Data of 17 patients were finally used for analyses (see S1 Fig for a flowchart depicting inclusion of participants). Thirteen neurologically healthy individuals matched for age and sex were recruited through community advertisement. The study was conducted in accordance with the standards defined in the Declaration of Helsinki and had been approved by the Ethics Committee of the University Medicine Greifswald (BB29/09b). All participants signed written informed consent forms and were financially compensated. The recruitment and experimental intervention started at 01.05.2015 and ended at 30.06.2019.

### Procedure and study design

The current examination was part of a larger study on emotion processing in stroke patients which contained a variety of examinations realized on three day within a week [20]. Hence, as data acquisition was realized after the beginning of the study a prospective observational study design was followed.

### Demographic and clinical characteristics

Age, handedness, and level of education (school years) were assessed via self-reports. Depression was tested with the Beck Depression Inventory II [31].

National Institute of Health Stroke Scale (NIHSS) scores assessed at admission to the hospital were available for 16 patients and applied as an indicator of stroke severity [32]. Clinical characteristics including diagnoses and age of lesion (in months) were taken from medical reports. To compare patients and controls, a Fisher’s exact test (handedness), a Chi-squared test (sex), and independent samples *t*-tests (age, school years, BDI) were computed. One patient missed two items of the BDI and, hence, was not considered for comparison.

### Neuropsychological tests

To rule out relevant cognitive impairments a neuropsychological evaluation was conducted which comprised verbal intelligence with the MWT-B [33], alertness with a simple reaction task [34], verbal comprehension with the Aachen Aphasia Test [35], verbal memory with the California Verbal Learning Test [36], executive functioning with the Trail Making Tests [37], susceptibility to interference with the German version of the Stroop Color-Word Interference Test [38], visuospatial memory with the Benton Visual Retention Test [39], and facial blindness with a computerized version of the Facial Identity Discrimination task adapted from the Florida Affect Battery [40] using pictures showing neutral facial expressions taken from the FACES database [41]. Group comparisons were realized with independent samples *t*-tests.

### Face processing task

For the face processing task in the MRI pictures of 10 face models (5 female, 5 male) showing emotional expressions were taken from the FACES database [41]. Following an established procedure [42] the selected faces were converted into gray scales, normalized in luminance and equalized in size. In addition, the faces were enclosed in an elliptic black frame to minimize the influence of expression-irrelevant features on task performance. The face processing task comprised two runs. During each run 5 blocks were presented which respectively showed 20 faces with the same expression (fearful, angry, disgusted, happy, or neutral) while each face model was shown twice. Each face was presented for 300ms and followed by a fixation cross (300ms) resulting in a total block length of 12s. Between blocks, a fixation cross was shown for a further 12s (see Fig 1A). Participants were randomly assigned to one of five stimulus orders created with the constraint that each expression was shown during the first and second run. Also, orders differed regarding the expression of the first block. The two runs were separated by a resting period (48s) that showed a fixation cross.

### (f)MRI imaging and analyses of (f)MRI data

MRI was performed using a 3T Siemens MRI (Magnetom, Siemens, Erlangen, Germany). The whole scanning lasted about 17 minutes. Information on imaging parameters have been provided in the Supplemental Information.

Also, details on preprocessing of fMRI and MRI data can be found in the Supplemental Information. Statistical analysis of the normalized fMRI data was performed using the SPM12 software (Wellcome Department of Cognitive Neurosciences, London, UK; www.fil.ion.ucl.ac.uk./spm) running under Matlab (Math Works; Natick, MA, USA). All events were modeled as blocked design with an event for each emotional expression (fearful, angry, disgusted, happy, neutral; containing 20 faces for each block; 2 blocks per emotion). As additional regressors realignment parameters were inserted and a high pass filter of 128s was applied as suggested in the default setting. Since recognition impairments in stroke cohorts have been reliably demonstrated for negative expressions [16, 17, 43] effects during these expressions were modeled as a first-level effect and used for group comparisons in a second level (t-tests: HC minus insula stroke; insula stroke minus HC).

Two ROIs were selected (see Fig 1B): The left fusiform face area (FG2_4; Anatomy Toolbox version 3.0); and the left ventral striatum (Oxford-Imanova Striatal Structural Atlas). Two different analysis procedures were performed: in a voxel-based ROI analysis effects between groups (HC minus insula stroke) were compared for significant effects within each ROI (small volume correction; p_FWE_<0.05) using standard SPM12 second-level statistics. In addition, we applied script-based ROI analyses extracting the highest betas per ROI for each participant and performing second level-group analyses with SPSS 21. Whereas the voxel-based analysis is optimized for effects in voxels with the same MNI-location between participants (optimal for ventral striatum) the second analysis allows for different locations of highest activated voxels between participants (e.g., optimized for different face areas between participants in the fusiform gyrus). Overall, both types of analyses should complement. Individual beta estimates for both anterior insulae were used for Pearson correlation analyses (total recognition accuracy, means of intensity ratings and SCR). Due to technical malfunction during transfer data was not available for one patient.

### SCR

SCR was continuously recorded during the face processing task in the MRI with two Ag/AgCl electrodes (4 mm diameter) filled with an electrolyte medium and placed adjacently on the hypothenar eminence of the palmar surface of the non-dominant hand. Electrodes were connected to a laptop via a GSR-MR module and a BrainAmpExG MR device. BrainVision Recorder software was used for recording the signal. The signal was sampled down to 10Hz using BrainVision Analyzer 2.0 (all Brain Products, Gilching, Germany). Ledalab 3.4.8 [44] was used to preprocess the signal using adaptive smoothing and to assess SCR as the average phasic driver (in μS). An analysis window of 0.9 s to 4 s after block onset was chosen since the first relevant rise in the signal toward stimulation typically occurs in that period [45] (see Fig 1C). The minimum amplitude threshold was set to 0.1 μS. A logarithmic transformation was carried out to normalize the distribution [46]. Mean values were then computed respectively for positive (i.e. happy), neutral and negative (fear, anger, disgust) facial expressions. A repeated measures (rm)ANOVA was computed with *Group* (HC, insula stroke) as between-subjects factor and *Block* (block 1 to 10) as within-subjects factor. A further rmANOVA was computed with *Group* as between-subjects factor and *Valence* (negative, neutral, positive) as within-subjects factor. As a profound habituation of the SCR over the course of the experiment was noticeable, a mean value was computed comprising the SCRs of the first three trials and compared between groups using an independent samples *t*-test. SCR amplitude is known to decline with age [47]. In the light of a large age span in our sample Pearson correlations were computed between age and total mean SCR (i.e. averaged across all blocks) respectively for both groups.

### Ratings of facial expressions

Accuracy of facial emotion recognition and experienced intensity had already been published together with lesion mapping and DTI analysis of tracts deriving from the insula with a paradigm using the same face stimuli and expressions in a sample of stroke patients including the here reported cases with insula lesions [20]. In this study pictures showing facial expressions were shown in a pseudo-randomized order with each trial consisting of a baseline phase (fixation cross, 3 s) and a viewing phase. After 6 s participants were asked to rate the nature and the intensity of the expression using forced-choice format scales additionally shown below the face for a self-determined duration. Twelve pictures were applied for each expression (six male and six female face models) resulting in 60 stimuli. Recognition accuracy and intensity ratings were re-examined for the here presented sample of patients with insula lesions using a rmANOVA with *Group* as between-subjects factor and *Valence* as within-subjects factor (factor levels see SCR).

In order to examine laterality of insula lesions further rmANOVAs were conducted similarly to those above, however with *Group* as between-subjects factor with three levels (HC, left insula lesion, right insula lesion). In case of significant main effects Bonferroni-corrected post-hoc tests were conducted. Additionally, recognition accuracy and mean intensity ratings including all expressions were applied for Pearson correlation analyses. All statistical analyses were performed using IBM SPSS Statistics 22 (Armonk, NY, USA). Plots were created using Microsoft Excel (Microsoft Cooperation, Redmont, WA, USA), R [48] and the ggplot2 package [49]. A final SPSS-table with data of the study can be downloaded on github: https://github.com/NitramNimod/face_localizer

## Results

### Group characteristics

Patients and healthy controls were comparable in all characteristics and neuropsychological capacities (Table 1). Also, no significant differences between patients with left and right insula lesions could be found. Lesion summation maps confirmed that lesions covered the insular cortex (S2 Fig).

**Table 1:** Participants’ characteristics.

	Healthy controls	Insula stroke	Statistics
**No. of participants**	13	17	-
**Age in years**	60.3 (14.4)	61.1 (13.8)	t = -0.157, p = 0.877
**Gender (F:M)**	7:6	6:11	χ^2^ = 1.033, p = 0.310
**Handedness (L:R)**	0:13	1:16	p = 1.0
**Months since stroke**	-	24.5 (28.3)	-
**Lesion volume (cm^3^)**	-	25.2 (34.0)	-
**NIHSS**	-	3.5	-
**School years**	10.3 (1.4)	10.1 (1.1)	t = 0.541, p = 0.593
**MWT-B**	28.9 (3.6)	27.6 (5.4)	t = 0.762, p = 0.452
**Simple reaction (ms)**	372 (68)	348 (84)	t = 0.825, p = 0.416
**AAT**	55.4 (4.5)	54.2 (4.4)	t = 0.736, p = 0.468
**CVLT**	52.2 (12.9)	48.8 (13.4)	t = 0.703, p = 0.488
**TMT B/A ratio**	2.5 (0.8)	2.6 (0.9)	t = -0.415, p = 0.681
**STROOP in s**	82.4 (19.1)	99.5 (26.0)	t = -1.992, p = 0.056
**Benton**	7.2 (1.4)	6.4 (1.5)	t = 1.402, p = 0.172
**BDI**	10.2 (8.1)	6.6 (6.1)	t = 1.392, p = 0.175
**FAB (%)**	95.4 (4.8)	94.1 (8.1)	t = 0.498, p = 0.622

Mean values are presented with standard deviation in brackets. F = female, M = male, L = left, R = right, NIHSS = Median score of the NIHSS at admission, MWT-B = number of correctly recognized words, AAT = score of the verbal comprehension task, CVLT = number of all correctly remembered nouns of the California Verbal Learning Task, TMT B/A ratio = TMT-B / TMT-A, STROOP = time required for the STROOP interference task, Benton = number of correct drawings, BDI = sum score of the Beck Depression Inventory II, FAB = Correct discrimination of facial identities.

### Face processing task

#### fMRI

**Fig 2:**
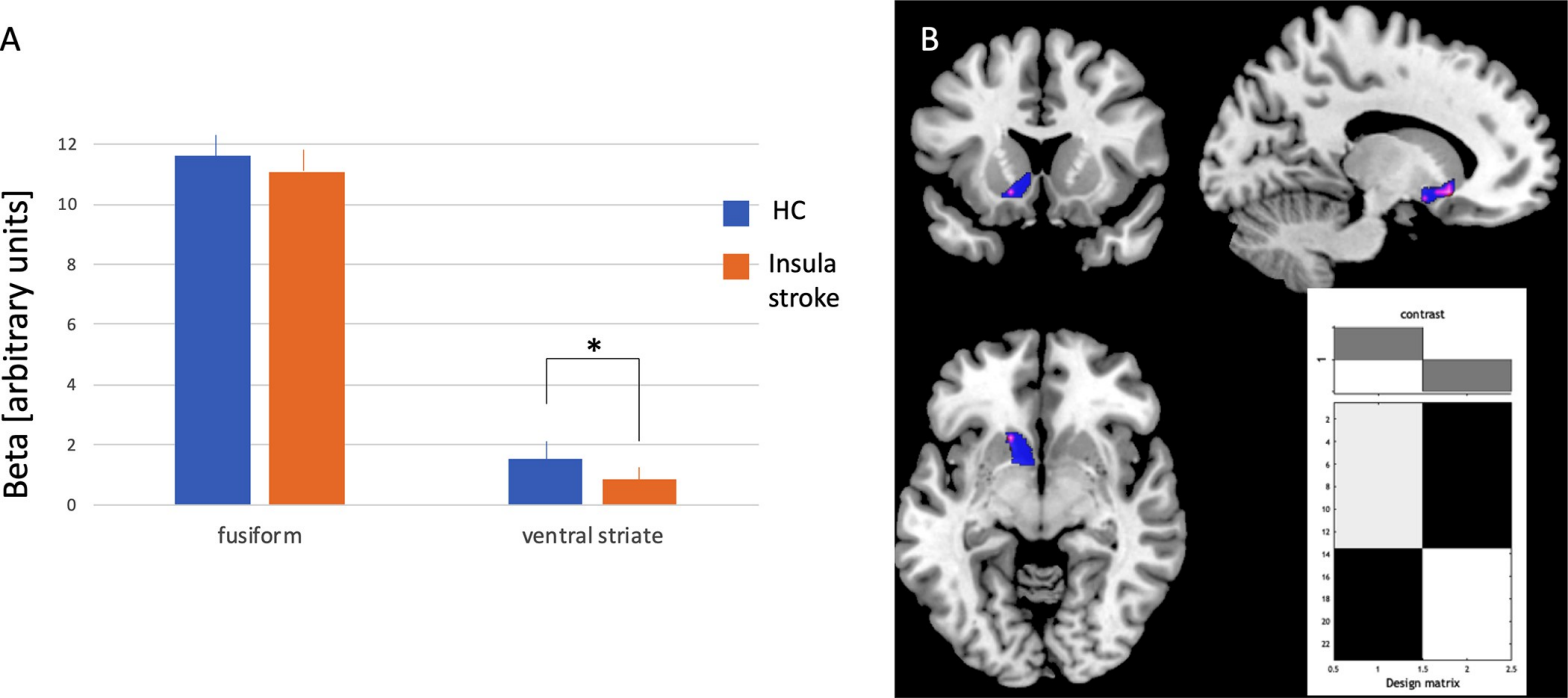
Results from both analyses strategies comparing BOLD-response to facial stimuli presenting aversive expressions. Bars represent means of beta estimates for the two ROIs for insula stroke patients and HCs (A). Hairs on bars provide standard errors. Analyses of BOLD activity of patients with left-hemispheric damage and HCs (B). Color-coded effects in red to orange and ventral striatum ROI in blue. Right bottom is demonstrating the second level group design. HC = healthy controls. Indicator of group difference: *p < .05.

Selection of beta-effects within ROIs revealed BOLD-differences between insula stroke and HCs for the left ventral striatum (beta for HC: 1.575±0.859, confidence interval (95%): 1.055–2.094; beta for Insula stroke: 0.873±0.768, confidence interval (95%): 0.463–1.282; t(27) = 2.322, p = 0.028) but not for the left fusiform face area (beta for HC: 9.705±4.252, confidence interval (95%): 7.135–12.274; beta for Insula stroke: 10.816±4.053, confidence interval (95%): 8.656–12.976; t(27) = -0.719, p = .479) (Fig 2A). The left anterior insula ROI, which served as a control area to test whether the predominantly lesioned region is also affected with decreased BOLD showed no relevant differences between groups (beta for HC: 2.769±1.703, confidence interval (95%): 1.740–3.798; beta for Insula stroke: 1.998±1.319, confidence interval (95%): 1.295–2.701; t(27) = 1.375; p = .18). Further analyses of patients with only left hemispheric damage demonstrated that left ventral striatum effect between groups was driven only by left hemispheric damaged patients (t = 3.74; p_FWE_ = 0.021) (Fig 2B). However, on a descriptive level, main effects were located more lateral superior to the ROI (main effect in ROI: -18, 4, -12; main effect in putamen: -21, 9, -12 with t = 4.22).

#### SCR

**Fig 3:**
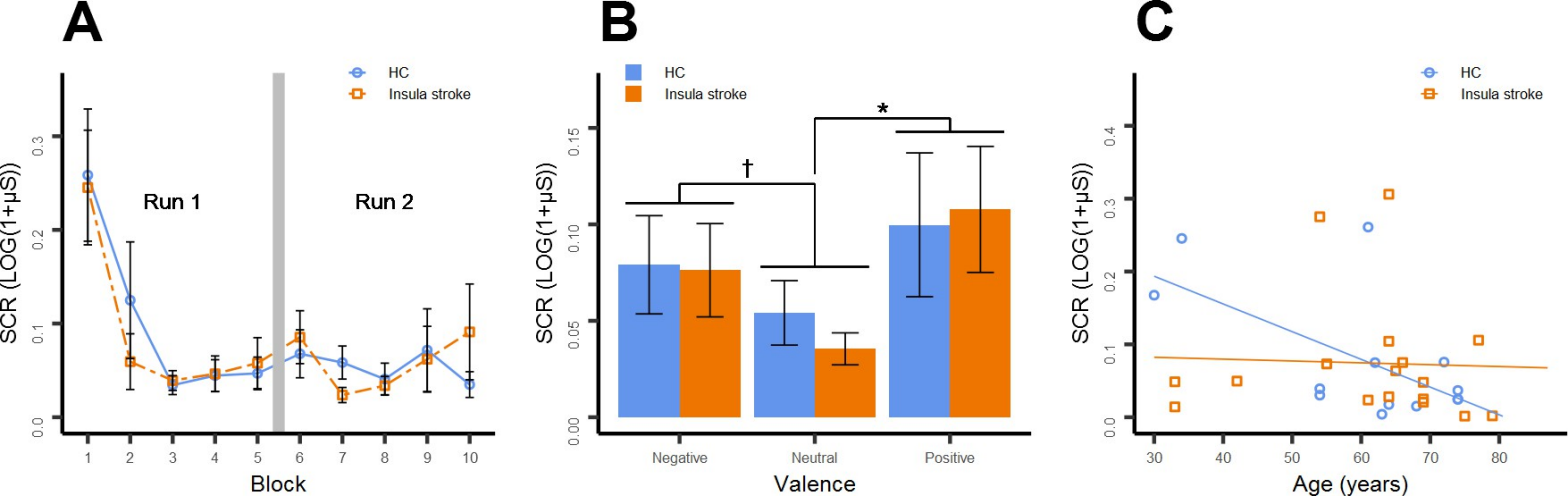
Mean SCRs for each block with a vertical column representing the pause between the two runs (A) and averaged for negative, neutral and positive facial expressions respectively for healthy controls and stroke patients (B). Correlations between age and total mean SCR for both groups with corresponding regression lines (C). Error bars represent standard errors of means. HC = healthy controls. Indicators of valence differences: *p < .05, †p < .1.

A decline in SCR over time was observable *(F*(9,252) = 11.140, *p* < .001) (Fig 3A). Post-hoc tests showed that SCR of the first trial was larger than SCRs of subsequent trials (*p*s < .05/.01) which did not differ from each other (*p*s = 1.0). No differences between patients and HCs could be shown (*(F*(1,28) = 0.014, *p* = .907). SCR differed between valences in both patients and controls *(F*(2,56) = 6.622, *p =* .003). Post-hoc tests showed that SCR during neutral expressions was smaller than SCR during negative (*p* = .051) and happy expressions (*p* = .014), while SCR during happy and negative expressions did not differ significantly (*p* = .343) (Fig 3B). No differences between HC and patients were found *(F*(1,28) = 0.020, *p =* .887). When considering lesion side no differences between groups were present (*p*s > .1).

No significant interactions between main factors could be found. Mean SCRs of the first three trials did not differ between HC and patients (*t*(28) = 0.464, *p* = .646; *F*(2,27) = 0.196, *p* = .823). A significant negative correlation between total mean SCR and age was found in healthy controls (*r* = -.618, *p* = .024), but not in insula stroke patients (*r* = -.040, *p* = .878) (Fig 3C).

#### Re-analysis of ratings of facial expressions

**Fig 4:**
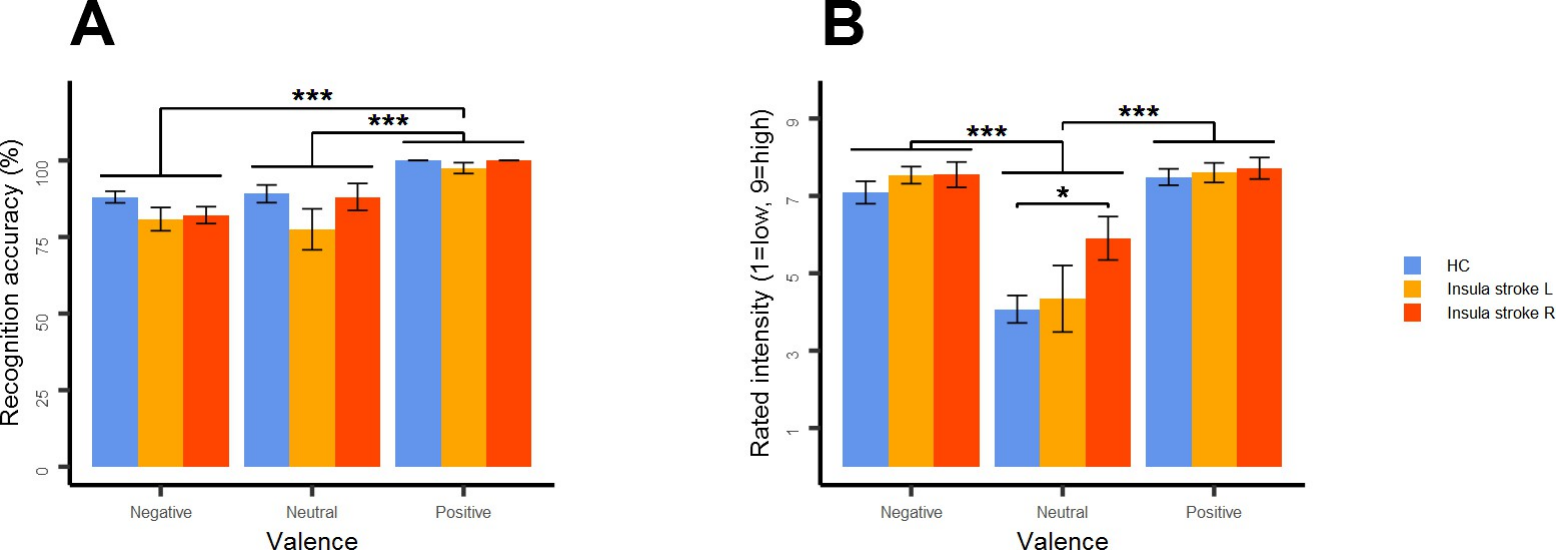
Mean recognition accuracy (A) and intensity ratings (B) for negative, neutral and positive facial expressions respectively for healthy controls and stroke patients. HC = healthy controls, L = left, R = right. Indicators of group and valence differences: ***p < .001, *p < .05.

Recognition accuracy differed between valences *(F*(2,56) = 22.565, *p* < .001) (Fig 4A). Post-hoc tests showed that positive expressions were better recognized than neutral and negative expressions (*p*s < .001). No differences between neutral and negative expressions could be found (*p* = 1). Patients showed a reduced recognition performance compared to HCs resulting in a trend significance *(F*(1,28) = 3.858, *p* = .060). When considering lesion laterality trend significant differences between groups were found *(F*(2,27) = 2.974, *p* = .068). Post-hoc tests showed that patients with left insula lesions had slightly poorer recognition accuracy than HCs (*p =* .067). Significant interactions between main factors could not be stated.

Intensity ratings differed between valences *(F*(2,56) = 64.488, *p* < .001) (Fig 4B). Post-hoc tests showed that neutral expressions were experienced as less intense than negative and positive expressions (*p*s < .001) while no differences between negative and positive expressions could be shown (*p* = .498). Patients and HCs did not differ in their ratings *(F*(1,28) = 2.147, *p* = .154). When considering lesion laterality no differences between groups could be stated *(F*(2,27) = 1.839, *p* = .178). An exploratory t-test showed that patients with right insula lesions experienced neutral expressions as more intense than HCs (*p* = .010). No significant interactions between main factors were present.

#### Association between fMRI activation, rating performance and SCR

**Fig 5:**
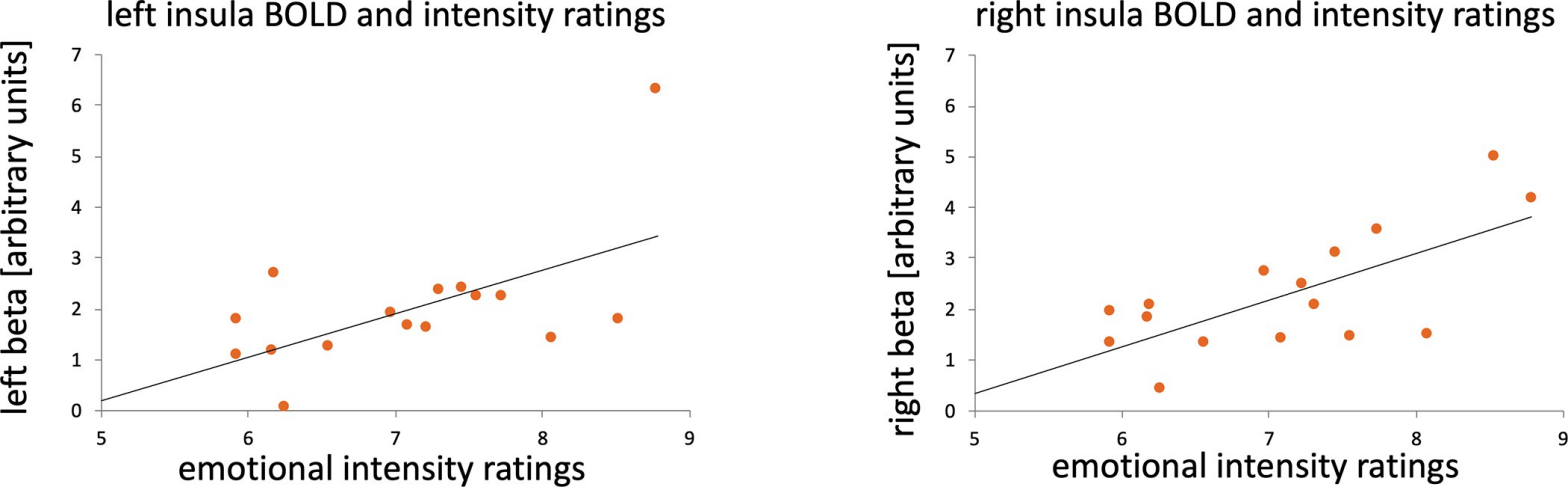
Correlations between beta values in both anterior insulae and emotional intensity ratings of facial expressions for stroke patients. Regression lines are plotted.

In patients, rating of intensity of facial expressions was positively associated with both left and right anterior insula fMRI-activation magnitude (left: r = 0.584; p = 0.018, right: r = 0.697; p = 0.003; Fig 5). Recognition accuracy, however, was negatively associated with left insula activation magnitude (r = -0.574; p = 0.02). There were no significant associations between SCR and insula activation magnitude.

## Discussion

### Main results- fMRI effect in ventral striatum (VS)

We here demonstrated that damage of the insula following stroke modulates left ventral striatum fMRI-activation magnitude to aversive emotional faces. For that we carefully selected a group of insula stroke patients who did not differ in a large battery of neuropsychological tests for visual, language and cognitive performance from healthy controls.

Other areas important for fMRI comparisons such as the left fusiform or the ventral striatum were not affected in our patient group as it could be verified by lesion mapping overlay. Functional imaging revealed no BOLD difference between groups in left anterior insula and left fusiform gyrus but a decrease in fMRI-activation in the patient group in VS. This could be verified for both fMRI-evaluation strategies–(voxel- and ROI-based). Whereas earlier face recognition processing activation in the fusiform gyrus was unaffected by lesion, those possibly modulated by insula function was decreased. This lack of effect in the earlier visual pathway speaks against an interaction of insula modulation of the early face recognition areas such as the fusiform gyrus as postulated before [50]. Since especially the anterior insula is densely interconnected with affective processing and awareness in the basal ganglia, temporal pole and amygdala, and orbitofrontal lobe [13, 14, 51] a crucial impact of a damage of this area might well effect functional processing in more downstream representation areas such as the VS.

### Skin conductance response

SCR differentiated between neutral and emotional expressions as similarly found previously in a healthy cohort [25]. Also, SCR amplitudes habituated already after the second block. In fact, habituation of SCR is a common finding [52] and also observable during the processing of facial expressions [53]. Contrary to our expectations, patients with insula lesions showed SCRs comparable with those of HCs. In contrast, in a number of different emotion-processing tasks, patients with insula lesions showed reduced SCR towards pictures of emotional scenes [28] and auditory stimuli [27]. An explanation might be that facial expressions induce lower subjective arousal [54] and smaller SCRs [55] compared to pictures of emotional scenes for example, and, hence, result in less variability in the data. What is more, identifying impairments in autonomic arousal might be further aggravated by a generally reduced response in older participants [50]. However, an absent correlation between SCR and age in our stroke cohort could reflect impaired autonomic arousal and was also reported in previous work on stroke patients [56]. Looking at individual values showed that a null correlation was primarily driven by lowered SCR amplitudes in the younger patients of our sample compared to HCs of a similar age. Interestingly, two of these young patients with lowered SCR had left insula lesions which could point to laterality effects.

### Re-analysis of ratings of facial expressions

The re-analyses of the data restricted to those patients with insula lesions showed again relevant impairments in emotion recognition and confirmed that happiness is the best recognizable expression [16, 17]. Though effects were not significantly lateralized impairments appeared more pronounced in patients with left insula lesions which is in line with the literature [19, 57] and which corresponds to the finding of lowered activation in the left VS. For example, the only patients who did not correctly recognize all happy expressions had a left insula lesion. Patients did not show reduced intensity ratings and experienced emotional expressions as more intense than neutral expressions as previously shown in a healthy sample [18]. Hence, sensitivity to facial expressions appears intact in our stroke cohort. Interestingly, patients with right insula lesions experienced neutral expressions as atypically intense. Similarly, higher intensity ratings, however, towards emotional expressions have been found in a patient with a lesion in the same area [57]. In the light of our data and previous work [18, 57], changes in experienced intensity (e.g. increased sensitivity) may not correspond to changes in recognition accuracy (e.g. increased recognition accuracy).

### Correlation analyses

Insula activation in patients was related to intensity ratings of facial expressions of emotions which could well be expected from a prior finding reporting associations between left insula BOLD and valence ratings [58] since intensity is closely associated with valence [59]. Hence, insula activation seems to reflect the emotional relevance of a stimulus (indicated by its rated intensity). However, we could not find lateralization effects as correlations were present for both insula cortices. A positive correlation between SCR and insula activity found in previous work [60, 61] could not be stated which might be again due to lowered autonomic response in our older stroke cohort and to the applied stimulus type.

Down-modulation of ventral striatum activation in our patients when compared to HCs might well be interacting with emotional valence and emotional facial recognition. Relevant interactions in association with dopamine availability had already been described in a small group of Parkinsonian patients with dopamine reduction and deficits in emotional valence processing of expressive gestures before [10]. However, the interaction between vascular BOLD and expression recognition in insula stroke remains challenging.

### Limitations

It could be argued that a decrease in ventral striatum BOLD response in the patient group is related to a possible lesion overlap with this area. However, the patients’ insula BOLD response was comparable with those of HCs despite lesions in that very structure. This suggests that the decreased BOLD in the VS is caused by decreased functional processing but not related to the mere structural damage.

The paradigm chosen here was optimized for localizing fusiform and occipital effects specific for faces but not for emotional limbic response. Other stimuli developing a more solid emotional response in a longer time frame might be even better suited such as videos of expressive gestures used before [6, 10].

In addition, patients with insula lesions are difficult to collect and recruit for such demanding imaging experiments resulting in the examination of only small samples which limits detection of effects to those with medium to high effect size.

## Conclusion

Our study showed that fMRI activation in an area directly interacting with the left insula in facial emotion processing was decreased in patients with left insula damage. In addition, fMRI activation in the anterior insula was positively associated with rated emotional intensity of stimuli. Besides lesion-symptom mapping and examination of white matter integrity changes in functional representation of emotion processing might be of relevance in further characterization of deficits following brain damage. Further work in similar patients might focus on functional connectivity changes and neurotransmitter decrease, especially dopamine.

## Supporting information

S1 Checklist Human participants research checklist. (DOCX)

S1 Fig Flow chart of participants. (DOCX)

S2 Fig Lesion distribution maps. (DOCX)

S1 File There are additional supplemental information for the methods. (DOCX)
