# Supplementary figures and images for: Down-modulation of functional ventral striatum activation for emotional face stimuli in patients with insula damage
Source: PLoS One. 2024 Jul 17;19(7):e0301940. doi: 10.1371/journal.pone.0301940 (PMC11253967; doi:10.1371/journal.pone.0301940)

Supplementary Figure 1:

***
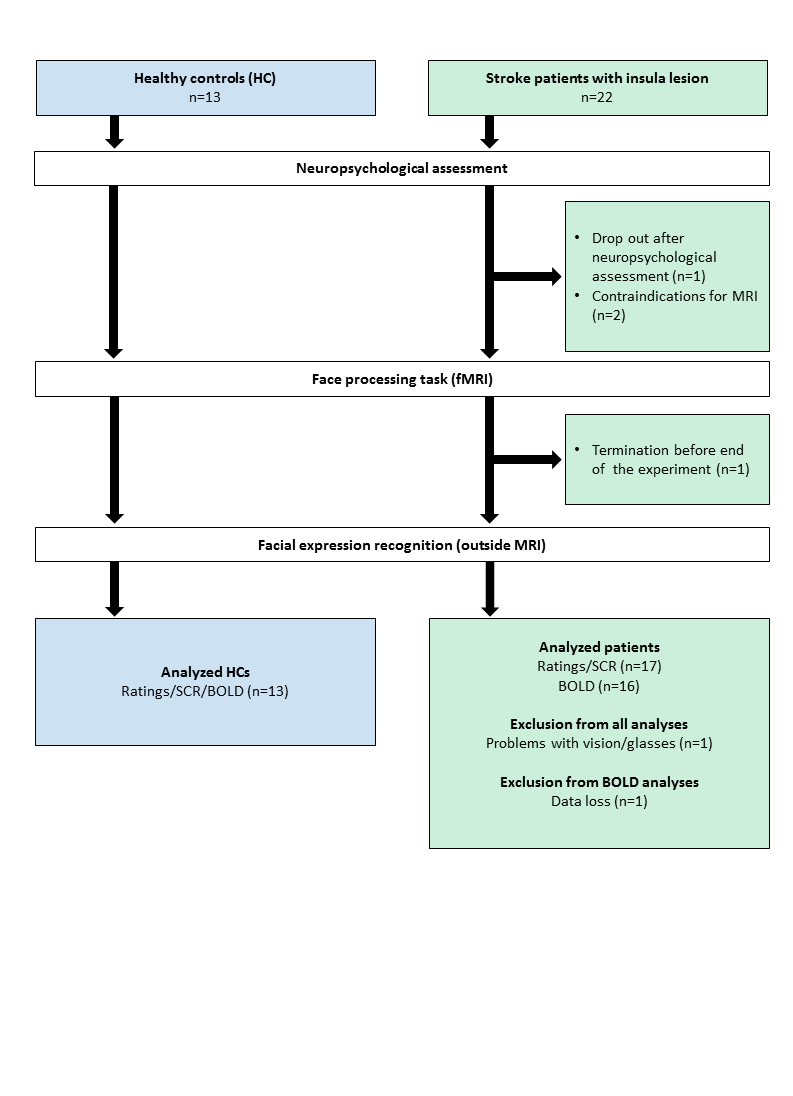
***

Supplement: S1 Fig — (DOCX) [file pone.0301940.s002.docx]

Supplementary Figure 2:


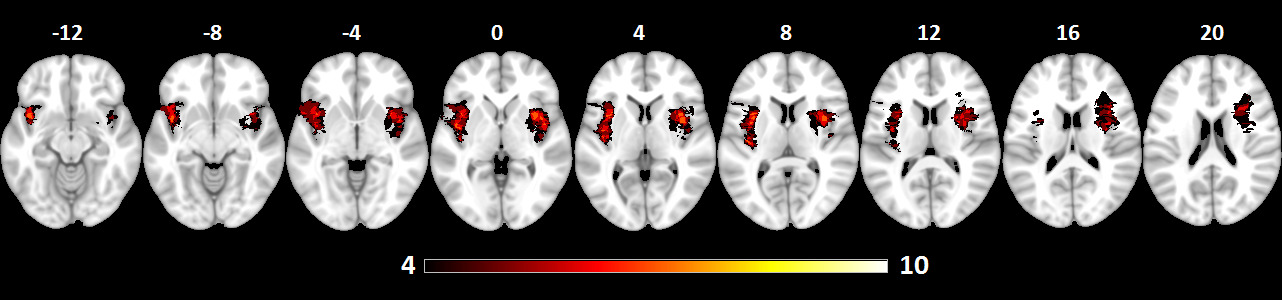

Supplement: S2 Fig — (DOCX) [file pone.0301940.s003.docx]
