# Supplementary material for: Down-modulation of functional ventral striatum activation for emotional face stimuli in patients with insula damage
Source: PLoS One. 2024 Jul 17;19(7):e0301940. doi: 10.1371/journal.pone.0301940 (PMC11253967; doi:10.1371/journal.pone.0301940)
Supplement: S1 File — (DOCX) [file pone.0301940.s004.docx]

**Supplemental Information for the Methods**

MRI:

Scanning was performed using a 3 T MRI equipped with a 32-channel head coil. During the functional experiment (face processing task), echo-planar images (EPI; TR 2000 ms; TE 23 ms; flip angle 70°; FOV 192 × 192 mm²; matrix size 104 × 104, voxel size 2 × 2 × 3 mm³, 1 mm spacing between slices) with 148 volumes with 33 slices were acquired. T1-structural scans were acquired (MP-RAGE, TR 1690 ms, TE 2.52 ms, flip angle 9°, matrix size 256 x 256, voxel size 1 × 1 × 1 mm³). The whole scanning lasted about 17 minutes.

Preprocessing:

MRI/ fMRI: Using structural T1-weighted images and MRIcron [43] lesions were manually drawn by an experienced neuroscientist and neurologist (M.L.). Lesion maps of stroke patients were then processed using Advanced Normalization Tools (ANTs, v2.2.043) to register and spatially normalize the T1-weighted images into MNI space (MNI ICBM152 6th generation). Individual lesion map images were transformed into MNI space by applying the same transformation using the GenericLabel-Interpolator. Lesion distribution maps were created using MRIcron and color-coded for overlap between participants (see Supplementary Figure 2). Functional imaging data were preprocessed using the Advanced Normalization Tools (ANTs, v2.3.5) [44]. The time course data were linearly registered to the average time series volume to correct motion-related misalignment. Although a gradient-echo-based field mapping was available, due to non-optimal image acquisition a non-linear approach via ANTs SyN (average fMRI time series image registered to T1w image) was chosen as it could be shown that only in the case of an optimal field map acquisition an unwarp procedure such as SPM’s Realign&Unwarp would produce adequate results [45]. Spatial normalization of the time course data into MNI template space was performed indirectly using the 3D-T1w structural image, and available binary stroke lesion maps were integrated into this process. Afterward, a Gaussian kernel with 8mm full width at half maximum (FWHM) was applied to the time course data to increase the signal-to-noise ratio and to fulfill the requirements for Gaussian Random Field Theory.
